# Manifestations and Potential Consequences of Information Distortion in Electronic Nursing Records: Qualitative Study

**DOI:** 10.2196/96679

**Published:** 2026-08-26

**Authors:** Jianan Wang, Yihong Xu, Wen Li, Zhichao Yang, Yushu Sun, Xiaoxiao Zhang, Hongying Pan

**Affiliations:** 1 Nursing Department, Sir Run Run Shaw Hospital, 3 Qingchun East Road, Hangzhou, Zhejiang, 310016, China, 86 13857188922

**Keywords:** electronic nursing records, information distortion, information ethics, data quality, nursing

## Abstract

**Background:**

Nursing records are a critical reflection of nursing practices and play a key role in ensuring the quality of patient care. However, these records sometimes fail to accurately represent the real-world health truth, resulting in information distortion. The manifestations and potential consequences of such distortion remain understudied, which may hinder efforts to improve nursing practices.

**Objective:**

This study aims to explore the primary manifestations and potential consequences of information distortion in electronic nursing records (ENRs).

**Methods:**

We conducted semistructured interviews at a Class-A tertiary hospital in China. A total of 12 nurses from various care units were recruited through purposive and snowball sampling. Inductive content analysis was used to analyze the qualitative data.

**Results:**

Qualitative analysis revealed that manifestations of information distortion in ENRs fall into 4 main categories: untimeliness, inaccuracy, incompleteness, and inconsistency. These categories are further subdivided into 7 subcategories: premature recording, delayed recording, incorrect data entry, fabrication, account misuse, missing data, and inconsistent data entry. The potential consequences of information distortion encompass 3 main categories: patient-related, nurse-related, and data-related. These categories are further subdivided into 5 subcategories: threats to patient safety, increases in negative emotions, damage to professional image, erosion of nursing professionalism, and impairment of secondary data use.

**Conclusions:**

The identified manifestations encompass a wide range of nursing activities, with potential consequences affecting patients, research, and caregivers. Strengthening ethics education, implementing monitoring mechanisms, establishing standardized guidelines, and leveraging technological solutions are recommended to reduce information distortion in ENRs. Further research on contributing factors and effective countermeasures is needed to mitigate information distortion in ENRs.

## Introduction

Health data reveal personal information about an individual’s past, present, or future physical or mental health status [1], playing a vital role in patient treatment, diagnosis, and decision-making [2]. Most health data are stored in health information systems and are reflected in medical and nursing documentation. Nursing documentation represents the care provided and the severity of a patient’s condition [3]. Proper documentation serves as a communication tool, quality assurance measure, educational resource, and research material [4,5]. In addition, it is a critical reference for doctors to understand the patient’s condition and take appropriate medical actions [6]. Therefore, nurses have a professional responsibility to ensure their records are complete and accurate [7,8].

However, discrepancies between nursing records and actual patient care can be problematic. Some research has found that certain nursing activities are performed but not documented [4,5,9]. De Marinis et al [10] observed that only 40% of nursing activities are recorded in nursing documentation, while Inan and Dinç [11] found that the consistency between actual nursing care and nursing records was only 77.6%. A study measuring health care data quality revealed that every 1% reduction in data completeness resulted in a 1.21% increase in missing events [12], highlighting the need to address data quality issues. In fact, the discrepancies in nursing records constitute some forms of information distortion, which occur when information documented in electronic nursing records (ENRs) fails to accurately reflect the underlying clinical reality [13]. Current studies focus primarily on constructing various data quality frameworks for health records [14,15]. Although information distortion is closely related to poor data quality and certain data quality dimensions (eg, incomplete documentation and documentation errors) overlap with manifestations of information distortion, it represents a broader ethical and behavioral phenomenon involving multiple forms of deviation and a wide range of contributing factors [16]. However, little research has explored information distortion in practical contexts. Existing studies have focused on isolated manifestations, such as insufficient documentation during nurse rounding or skin assessments [4,5]. A comprehensive understanding of the full spectrum of information distortion in nursing records and its potential consequences is lacking, limiting the development of effective strategies to prevent and mitigate information distortion.

Nursing records can be in paper or electronic formats. With technological advancements, ENRs have become prevalent in health care settings, often replacing paper records either partially or entirely [17]. Therefore, it is crucial to adapt to these changes and explore the manifestations of information distortion within the context of ENRs.

Although China’s efforts in informatization started later than those of high-income countries, the Chinese government has actively promoted informatization through policies such as the 14th Five-Year Plan for National Informatization and the High-Quality Development Promotion Action of Public Hospitals. In recent years, the integration of advanced information technologies such as 5G, big data, and the Internet of Things into Chinese hospitals has expanded significantly in both breadth and depth [18]. According to the National Health Commission, the health information technology development index in China reached 74.14 in 2024 [19]. Data from the China Hospital Information Management Association also show that nearly 100 hospitals in China have achieved level 5 or higher in electronic medical record (EMR) adoption. The implementation of information systems such as clinical decision support systems, hospital information systems, EMRs, and picture archiving and communication systems has optimized clinical data management and improved the quality of health care services [20,21]. With the rapid growth of informatization, it is crucial to address information ethics issues to support the sustainable development of these advancements. Examples of such issues include information disclosure, distortion, alienation, and injustice. Notably, information distortion compromises data accuracy, which can directly jeopardize patient health and safety, making it a critical area that requires focused attention [13].

Therefore, this study aims to explore information distortion from a Chinese perspective, specifically examining its manifestations and potential consequences in ENRs. The goal is to enhance the understanding of information distortion and its implications for nursing practice and patient care within the Chinese context.

## Methods

### Study Design

When exploring social phenomena, qualitative research is suitable [22]. Therefore, a qualitative descriptive design was employed. Semistructured interviews were chosen as they allow for flexible yet guided questions, facilitating an in-depth exploration of participants’ thoughts and experiences [23]. All interviews were conducted face-to-face. This study also adhered to the COREQ (Consolidated Criteria for Reporting Qualitative Research) guidelines [24] (Checklist 1).

### Setting

The study was conducted in a Class-A tertiary hospital in Zhejiang Province, which has been using electronic documentation for nearly a decade. The hospital has over 20 years of experience with electronic documentation and integrated a nursing decision support system into its nursing information system in 2016. Its advanced level of digitalization has provided nurses with a comprehensive technological environment, equipping them with extensive experience in using the system. This experience is invaluable for gaining insights into issues related to information distortion.

### Recruitment

The interviewed nurses were recruited using purposive sampling and snowball sampling. To gain deeper insights into the complexities of information distortion, we chose to invite members of the Information Committee to participate in the interviews. Each department has a representative in the committee. These committee members are nurses who are actively engaged in clinical practice while also assuming additional roles, such as addressing system usage concerns and sharing technical expertise with their colleagues. They interact with information frequently, making them more likely to have a deeper understanding of the phenomenon of information distortion. We explained the basic concept of information distortion and the objectives of the research to committee members, allowing them to decide freely whether to participate. Since information systems differ across specialties, we used maximum variation sampling based on departmental categories to ensure that participants represented a broad range of clinical nursing practices. Additionally, committee members were invited to recommend other nurses who had been involved in clinical informatics-related work or projects as potential interviewees. The inclusion criteria for nurses were as follows: (1) clinical registered nurses employed at the hospital for at least 1 year and (2) voluntary participation with signed informed consent. Nurses from other hospitals who were present for training, as well as those absent during the study period (eg, on leave, on vacation, or attending external training), were excluded.

### Data Collection

A researcher (JW), a female PhD student trained in qualitative methods, conducted a total of 12 face-to-face semistructured interviews between January 5, 2024, and April 8, 2024. The interviews were conducted in Chinese, either in a coffee shop or in a demonstration classroom within the hospital. Data saturation was reached when no new insights emerged during the interviews. No other individuals were present besides the participants and the interviewer. The interviewer had no prior relationship with the participants, which helped reduce biases and potential ethical concerns. As part of the interview process, participants completed a short questionnaire about their personal characteristics, including gender, age, education, position, professional title, work experience, and work department. A topic guide with open-ended questions (Multimedia Appendix 1), developed through a review of existing literature and expert opinions and tested by 2 nurses beforehand, was used to cover all relevant topics during the interviews. Participants were asked to describe instances of information distortion they had experienced or observed in their clinical practice, as well as the potential consequences, including those they had witnessed among their colleagues. The interviewer took field notes during the interviews to enhance the data and mark key moments [25]. Interviews lasted between 35 and 91 minutes and were recorded with the written consent of the participants. The audio recordings were transcribed into Chinese by the researcher (JW) and then verified by the participants. No repeat interviews were conducted.

### Data Analysis

Inductive content analysis was conducted to analyze the qualitative data, following 3 phases: preparation, organizing, and reporting [26]. Two researchers (JW and YX) independently read the transcripts line by line, noting as many headings as necessary to capture all aspects of the content [27], using NVivo 12 (QSR International Pty Ltd). The headings were compiled into coding sheets, and categories were generated and supported by quotations derived from the interviews. The 2 researchers then compared and discussed each code and category, refining them and adding relevant codes and potential categories as needed. Participants also received a summary of the identified categories along with their own quotations for review, offering them an opportunity to provide feedback on the accuracy of the findings in reflecting their experiences. Finally, the entire research team reviewed and discussed the identified categories and subcategories to ensure coherence.

Since this study is presented in English, analyzing data in Chinese might raise concerns regarding trustworthiness. To address this, a qualified translator first translated the initial transcripts into English. During the process of identifying categories, subcategories, and representative quotations, the bilingual authors (JW and YX) carefully reviewed and adjusted the translations to ensure they accurately captured the intended meanings within the Chinese context in nursing. The other authors then independently reviewed the content for accuracy and provided feedback.

### Rigor

The rigor of this study was ensured by the trustworthiness of the data (credibility, transferability, dependability, and confirmability) [28]. Credibility was ensured by having 2 researchers independently code the data, followed by discussions within the research group to resolve any ambiguities or discrepancies. Furthermore, member checks were conducted by inviting participants to review and validate the identified categories and subcategories. Transferability was ensured by providing sufficient contextual information about the information environment in China, enabling readers to relate the findings to their own positions. Dependability was ensured through a detailed description of the participants’ inclusion and exclusion criteria, data collection and analysis procedures, and participant characteristics. Finally, confirmability was ensured through regular reflection on the researchers’ biases and a commitment to transparency and openness. The researchers also received comprehensive training in qualitative research methods, equipping them with the essential skills and knowledge needed to conduct the study reliably.

### Ethical Considerations

This study is part of a larger research project, and ethical approval was obtained from the Ethics Committee of Sir Run Run Shaw Hospital, Zhejiang University School of Medicine (approval number 2024; research number 0270). Prior to the interviews, each participant signed a written informed consent form after full disclosure of the study’s aim. All participants were informed that they could withdraw from the study at any time without any obligation. Data analysis and reporting were conducted anonymously to protect participants’ privacy.

## Results

### Participant Characteristics

All participating nurses were women, with an average age of 32 (SD 3.5, range 26‐37) years and work experience ranging from 4 to 14 years. They worked in various nursing units, including internal medicine, surgery, and the intensive care unit. Participant characteristics are further described in Table 1.

**Table 1.** Characteristics of participants (N=12).

Characteristics	Values, n (%)
Sex	
Female	12 (100)
Age (y)	
25‐29	3 (25)
30‐34	5 (41.7)
35‐39	4 (33.3)
Education	
Bachelor’s degree	10 (83.3)
Master’s degree	2 (16.7)
Post	
Clinical nurse	8 (66.7)
Advanced practice nurse	2 (16.7)
Nurse educator	2 (16.7)
Professional title	
Primary nurse	3 (25)
Charge nurse	9 (75)
Work experience (y)	
1-5	2 (16.7)
6-10	6 (50)
11-15	4 (33.3)
Work unit	
Internal medicine	7 (58.3)
Surgery	4 (33.3)
Intensive care unit	1 (8.3)

### Manifestations of Information Distortion in ENRs

#### Overview of Manifestations

**Figure 1.**
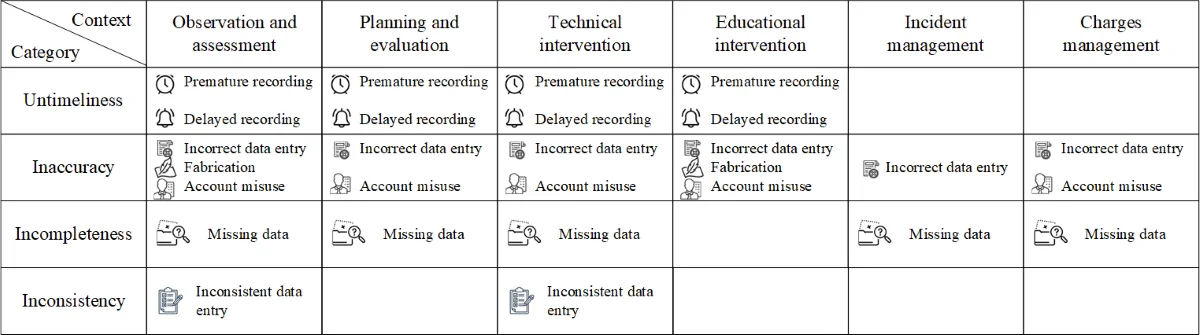
Manifestations of nurses’ information distortion in electronic nursing records.

The content analysis revealed that manifestations of information distortion in ENRs fall into 4 main categories: untimeliness, inaccuracy, incompleteness, and inconsistency. Seven subcategories were derived: premature recording, delayed recording, incorrect data entry, fabrication, account misuse, missing data, and inconsistent data entry. In addition, we identified specific nursing contexts in which these manifestations are more likely to occur, including observation and assessment, planning and evaluation, technical intervention (preventive and curative), educational intervention, incident management, and charges management. The findings are synthesized in Figure 1.

#### Untimeliness

This type of manifestation occurs when nurses write ENRs at inappropriate times. This category is subcategorized into 2: premature recording and delayed recording.

##### Premature Recording

Nurses indicated that they would record times before the events actually occurred. Nurses stated that they might complete the assessment forms and nursing care plans before actually checking patients.

*Some people have a habit of copying the assessment forms and care plans for their patients in advance, then doing rounds to check the patients. Then, they go back and make changes. With so many patients, it could take an hour to finish, but they don’t change the recorded time on the computer*.

[N3]

Another nurse stated that she recorded the time earlier than the actual time when performing interventions, such as infusing blood:

*We have drip rate requirements for blood transfusions, for red blood cells, it’s 60 drops per minute, and there’s a formula to figure out when it should be done. But sometimes, with all the factors affecting the tubing, it’s impossible to meet that exact time, so I’ll close the loop in the PDA (Personal Digital Assistant), you know, I have to mark it as finished in the record, even though it’s still infusing.*

[N7]

Nurses also stated that the education time was sometimes earlier than the actual time:

*We sometimes check off the education part when the medical order is issued, then give the education, like providing education when we’re actually giving out the medication. So the timing isn’t quite right*.

[N5]

##### Delayed Recording

Nurses mentioned that they might also record event times later than they actually occurred. A nurse said that they might forget to assess or give care plans and make up for it later:

*There are so many things, so sometimes I forget to update the assessments or the care plans. Then, right before leaving, I’ll check and go, ‘Oh no, I missed that’, and quickly fill it in*.

[N1]

Nurses also explained that this happens during curative interventions. When they administer medication while awaiting a delayed doctor’s order, they give the medication first and document the time afterward to comply with regulations. One nurse said the following:

*We (with doctors) agreed to administer 8 units of insulin, but the doctor’s order only specifies 6 units. We might ask the doctor to update the order to add the extra 2 units later. However, you might end up administering the insulin before the order is updated and yet the time on your records looks perfect*.

[N3]

In educational terms, in addition to premature recording, nurses mentioned that they might sometimes delay recording:

*When the meds are prescribed, they pop up one by one, and I can’t just sit there and watch each one to check it off, right? So I’ll catch up on it later. Like, I might go through all the education items from the morning at noon. I don’t care what time they were ordered—7, 8, 9, or 10 o’clock—I just check them all off at once*.

[N2]

### Inaccuracy

This type of manifestation indicates that the contents of ENRs cannot reveal the truth. This category is further divided into 3 subcategories: incorrect data entry, fabrication, and account misuse.

#### Incorrect Data Entry

There are times when events described in ENRs do not accurately reflect the truth. When assessing the patient’s condition, some issues may arise, such as errors in location. Some nurses noted:

*Once during a tube assessment, when I took over from another nurse, I found out that for nearly half a month, the record said the tube was in the left nostril when it was actually in the right one*.

[N1]

Nurses also mentioned that they would intentionally assess the patient’s condition as more severe to alert doctors:

*To use restraints, we need a doctor’s order. Before the doctor gives the order, the patient’s risk score has to be pretty high. So we might rate them higher than actual to make sure they get the restraints*.

[N12]

Nurses also found that the nursing care plans they create are sometimes not suitable for patients, and their evaluation faces similar issues. One nurse noted:

*In fact, some of patients even know more than we do, but we still put down “knowledge deficit” (when making the nursing care plan)…While the patient is in the hospital, it’s rare for us to mark nursing evaluations as “completed.” We usually just keep marking ‘partially completed’ until they’re discharged, even if they no longer have the issue*.

[N3]

When performing preventive interventions, nurses mentioned that they choose to continue providing nursing care for patient safety even after the order had been discontinued:

*For example, at 9 o’clock, the doctor’s restrictive order expired. We documented in our nursing records that we stopped the restriction, but in reality, we kept enforcing it to keep the patient safe, without asking the doctor to renew the order*.

[N12]

Educational templates offer convenience by significantly reducing nurses’ workloads. However, they can lead to errors if nurses fail to apply their own judgment. One nurse said the following:

*We have a template for VTE (venous thromboembolism) discharge education, it should be adjusted based on the patient’s needs. However, we often use it without any change…Like the template suggests quitting smoking but the patient doesn’t even smoke.*

[N6]

Additionally, nurses mentioned that when they have reported an incident, such as a pressure ulcer, they may not be able to confirm that it has healed. One nurse said:

*I think pressure ulcers are also a problem. We may think they have healed, but the assessment forms are still filling up, no one records “healed.”*

[N6]

Regarding charges, nurses admitted that they sometimes intentionally overestimate a patients’ condition to increase their incomes:

*We sometimes score the Braden scale at 18 or below on purpose so we can charge for it, as only scores of 18 or below qualify for high-risk pressure ulcer fees. For some patients who have undergone ERCP (Endoscopic Retrograde Cholangiopancreatography), their actual scores might be above 18, but we score them lower to fit the charging criteria*.

[N1]

#### Fabrication

There have been instances in which nurses documented tasks in ENRs that were not actually performed. Under the pressure of excessive workloads, some nurses may resort to fabricating their observation records for convenience:

*For critical care charts, we have to record data every hour. Sometimes if we’re really busy and the patient seems fine, we’ll just fill in that hour based on other times*.

[N9]

*If I’m in charge of rooms 9 to 16, I’ll record the same position for everyone in those rooms at a certain time. Like at 10 o’clock, they all ‘left side’. And at 12 o’clock, they all ‘right side.’*

[N4]

Moreover, nurses highlighted that fabrications are more common in their education records:

*Education problems are pretty big. When handing out oral medications, there are too many medications, like a dozen or more. We might miss the education step, we don’t explain what the new medications are for, we just focus on checking the patient’s identity, even though the records show that we provided the education…We just don’t have the time or energy to explain what the basic prevention measures for VTE include and what needs to be done. But the records do show that we provided education on VTE prevention*.

[N2]

#### Account Misuse

Nurses have their own accounts on ENRs and personal digital assistants, and misuse of these accounts can sometimes occur. One nurse said:

*Some people don’t log out of the PDA after their shift, and sometimes I don’t check carefully. So I might use the PDA all day with the previous shift’s account. All the charges and records for the day end up under their name.*

[N11]

The hospital has specific rules for correcting records, allowing only nurse leaders to make changes after 24 hours have passed. However, nurses often use their leaders’ accounts to make corrections without informing them.

*We all know our leader’s code. Sometimes, if there’s a mistake in the record, we use the leader’s code to make corrections.*

[N4]

### Incompleteness (Missing Data)

This type of manifestation indicates that the content of ENRs is less comprehensive than the actual nursing activities performed. This category consisted of a single subcategory: missing data.

Nurses stated that they tend to omit records either intentionally or unintentionally. One nurse mentioned that she did not record her thoughts during assessment because her experienced colleagues did not write them down:

*They (experienced nurses) didn’t write it, so maybe it’s not necessary*.

[N3]

Nurses also stated that new nurses are especially more likely to miss data. One nurse described this issue when discussing the nursing care plan:

*Some less experienced nurses, for example, might focus all their efforts on bringing down a patient’s fever after the patient has had it for a day. But they might forget to include the issue of the high temperature in their documentation*.

[N8]

When performing technical interventions, nurses also acknowledged that they sometimes omit certain details from documentation.

*When an infusion gets interrupted, you usually don’t really pay attention to it. If a patient needs to go for a test and there’s half a bag of saline still hanging, you won’t remember to mark it as interrupted*.

[N2]

Incident management helps prevent error recurrence and improve care quality [29]. However, nurses tend not to report medication errors:

*In our oncology unit, patients receiving chemotherapy also get an anti-nausea medication like ondansetron every 8 hours. For instance, if it’s given around 11 or 12 in the morning, we prioritize using the gastroprotective medication before chemotherapy. But with so many other medications to give throughout the day, sometimes as late as 10 or 11 at night, the timing for that small dose of ondansetron gets pushed back. It’s actually a near miss, but we don’t report it.*

[N6]

When it comes to charges, nurses stated that their established work habits sometimes lead to omissions in documentation.

*Our work habit is to charge for services in the morning. So, if a nebulizer treatment starts around noon, we won’t charge for that day, just once. In this case, a mask is given for that day, but no fee is collected.*

[N1]

### Inconsistency (Inconsistent Data Entry)

This type of manifestation refers to contradictions in data within ENRs. There may be disagreement between data in ENRs or between ENRs and another data source such as EMRs. This category consists of one subcategory: inconsistent data entry.

The lack of interoperability could hinder health professionals from exchanging information. Nurses stated that insufficient integration of information systems across different departments or systems could lead to inconsistent data in assessment. The lack of standard medical codes could lead to data recorded about the same patient having different descriptions:

*This patient was transferred from the ER (Emergency Room) to our ward, but I can only find his medical records by checking the discharge notes. If I look in our system, there’s nothing there, it’s like I’m dealing with a new patient*.

[N1]

*There are situations where the doctor and we nurses both assess the patient, but we record different chief complaints. The doctor might write “diagnosed with pancreatic cancer over seven months ago,” while we write “a few days post-chemotherapy.”*

[N7]

In addition, the introduction of data capture technologies, which make nursing practices more labor-saving and effective, can sometimes also lead to inconsistencies with manually entered records. Nurses expressed this issue during curative interventions:

*If a patient’s oxygen saturation drops, we handle the situation first and document it later. But vital signs are updated periodically, so while you might note the low oxygen levels, the recorded vital signs at that time could show normal, creating an inconsistency between the two*.

[N7]

### Potential Consequences of Information Distortion

#### Overview of Potential Consequences

**Figure 2.**
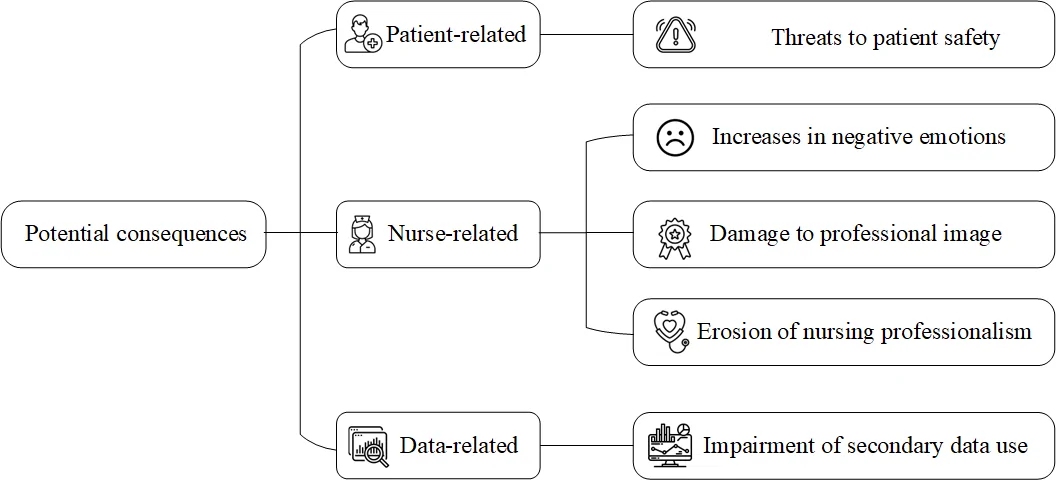
Potential consequences of information distortion in electronic nursing records.

The data indicated that the possible consequences of information distortion could be classified into 3 categories: patient-related, nurse-related, and data-related. These can be further divided into 5 subcategories: threats to patient safety, increases in negative emotions, damage to professional image, erosion of nursing professionalism, and impairment of secondary data use. The findings are synthesized in Figure 2.

#### Patient-Related: Threats to Patient Safety

Nurses indicated that failing to document changes in a patient’s condition could impact both nurses’ and doctors’ judgments about the patient. Inaccurate or omitted documentation may obscure the patient’s true clinical status, delaying the recognition of deterioration and the timely escalation of care, which may ultimately result in delayed treatment. Furthermore, distorted documentation may contribute to medication errors by leading clinicians to misjudge the patient’s condition and make inappropriate clinical decisions, thereby posing significant risks to patient safety.

*We often rely on the critical care sheet or assessment sheet to track significant changes in a patient’s condition. If our previous records aren’t accurate, it will definitely affect how we judge the patient’s condition later, right? We wouldn’t know when the changes actually started, and this could throw off our focus*.

[N10]

*If a patient’s blood pressure reads 180 but he’s shivering, and you only record the high blood pressure and inform the doctor, the doctor will trust that data and might prescribe other medications. If they prescribe a blood pressure med, like oral nitroglycerin, and you give it to the patient who’s actually shivering, which could mean they have an infection, right? If they’re in septic shock, their blood pressure would already be dropping. Giving them a blood pressure medication could be really dangerous*.

[N5]

#### Nurse-Related

##### Increases in Negative Emotions

Nurses expressed that the appearance of information distortion could make them feel regret or upset, intensifying their negative emotions.

*(If I don’t have the information distortion) I won’t be regretting at home that I didn’t write accurate records, thinking about what I should have done at that time.*

[N6]

*If they find my record is distorted, I’ll definitely feel a bit down about it. I mean, who doesn’t want their records to be perfect?*

[N12]

##### Damage to Professional Image

When information in the records does not match the actual situation, the likelihood of medical disputes may increase, and patients’ trust in nurses may decline, affecting nurses’ professional image.

*If there’s a problem with the drug’s expiration and patients notice it, especially if it says something like “12 hours”, it can cause a medical dispute cause issues. And patients might feel they can’t fully trust we nurses, thinking, ‘How could they make mistakes on something like this?’*

[N8]

##### Erosion of Nursing Professionalism

Constantly distorted information can desensitize nurses, causing them to overlook important possibilities. This can erode nursing awareness and expertise, ultimately undermining the thoughtful and conscientious spirit essential to the profession.

*Many patients, including those who’ve had surgery, often have a care plan for a long time before they’re discharged. We rarely make any changes, so there’s hardly any real awareness of their care needs.*

[N4]

### Data-Related: Impairment of Secondary Data Use

Secondary data use refers to the use of data for purposes other than those for which it was originally collected, such as quality improvement or research analysis [30]. As ENRs become increasingly prevalent in clinical practice, secondary analysis of these data is also commonplace. In terms of incident management, nurses expressed that distorted information about the cause of an incident can introduce bias in quality improvement, leading to ineffective improvement measures. As a result, incidents may continue to occur, rendering significant efforts futile.

*Because this (incident reporting) involves the subsequent analysis, why did this unexpected event happen? Were your measures effective? I think it is necessary to restore the actual situation. For example, if I say I put a restraint mitt on the patient but they still pulled out the tube, during analysis, wouldn’t they think that even with the mitt, the patient could pull out the tube? But if the mitt was not actually used, then maybe if it had been, the patient wouldn’t have pulled out the tube. Without an accurate record of the real situation, you can’t analyze the reason properly*.

[N12]

From a research perspective, nurses noted that distorted records could undermine the validity of findings generated from such data, ultimately compromising the reliability of scientific research.

*If I’m working on a pressure ulcer risk prediction model, things like how long a patient stays in bed or how often their position is changed are key factors. If those records aren’t accurate, they will affect the model’s accuracy. In that case, what’s the point of developing such a model?*

[N7]

## Discussion

### Principal Findings

This research is the first to clearly identify manifestations of information distortion in ENRs. Although previous studies do not explicitly use the term “information distortion,” some specific manifestations of distortion have been explored, including failure to record in a timely manner [31,32], inaccurate data [30,33], nursing practices performed but not recorded [4,5], and discrepancies in the same information [34]. These findings are related to our results, which include manifestations of delayed recording, incorrect data entry, missing data, and inconsistent data entry. Our study further contributes by adding manifestations of premature recording, fabrication, and account misuse within the context of Chinese nursing. Some of the identified manifestations raise ethical and professional concerns, as they may undermine the principles of accountability, responsibility, and expertise outlined in the International Council of Nurses Code of Ethics for Nurses [35]. Structured ethics education and training are needed to strengthen nurses’ awareness of accurate and responsible documentation practices. In addition, real-time documentation monitoring systems could be developed to provide oversight and support early identification of documentation-related issues. However, it is important to recognize that not all instances of information distortion represent intentional misconduct. For example, some behaviors identified in this study, such as overassessment and continued restrictions, were driven by nurses’ intentions to enhance patient safety, even when this required them to assume additional responsibilities. Therefore, considering the complexities of the clinical context in which these behaviors occur, it is inappropriate to see the issues in black and white.

Another important finding is that manifestations of information distortion permeate almost every facet of nursing practice, which is concerning. Previous studies have explored manifestations of information distortion in various nursing contexts, including assessments, evaluations, technical and educational interventions, and incident reporting [5,10,36]. Our study expands on this by identifying information distortion in nursing care plans and charge management, which may be related to the hospital’s comprehensive electronic nursing systems, which provide nurses with more intuitive user experiences. In our interviews, participants from every department reported experiencing or witnessing information distortion in ENRs, suggesting that this issue is not limited to specific departments, such as those that are extremely busy or poorly managed. Considering the widespread occurrence of information distortion, standardized documentation guidelines tailored to different nursing contexts are needed. In addition, review and feedback mechanisms should be established not only through hierarchical supervision but also through peer-to-peer feedback to improve documentation quality.

We also found that the potential consequences of information distortion in ENRs affect patients, caregivers, and the secondary use of data. Some studies on data quality have pointed out that incomplete or inaccurate data can put patients’ health care at risk [37] and affect the reuse of data for analysis [38,39], which is consistent with some of our findings, including threats to patient safety and impairment of secondary data use. Our research further explores the nurse-related potential consequences, offering an example of the impact of information distortion on health care professionals—an area seldom investigated in previous studies. Future studies could further develop and refine frameworks for understanding potential harms associated with health data based on these findings [40].

Although the issue of information distortion has existed since the era of paper-based records, manifesting in behaviors such as reluctance to report incidents or falsification of records, some of the manifestations identified in this study suggest that advancements in information technology may be influencing this phenomenon. Examples include the “copy then adjust” habit, the inappropriate use of templates, and the misuse of accounts, which may align with Burnum’s perspective that the development of health information technology, such as electronic health records, has not improved the quality of recorded data; instead, it has resulted in the recording of a larger amount of poor-quality data [41]. However, the trend toward greater digitization is irreversible. Given the pervasive manifestations of information distortion and the severe potential consequences, further research is needed to explore the influencing factors and develop strategies to address information distortion in ENRs, with the ultimate aim of enhancing the quality of records. Our research has highlighted factors such as heavy workload, years of experience, lack of interoperability, and the absence of standardized medical codes, which align with existing literature [34,42]. Potential countermeasures, such as the adoption of AI scribes and improvements in interoperability among electronic health record systems, could be considered to reduce information distortion [43]. Further research is needed to explore more related factors and countermeasures based on our findings.

### Limitations

This study has several limitations. Although the sample size may appear small, data saturation was achieved, which is consistent with the typical range of 12 to 20 interviews required for qualitative research [44]. Although nurses were recruited from diverse departments, the study was conducted in a single hospital in China, and the findings may have been influenced by its specific organizational and cultural context. Nevertheless, this study provides valuable exploratory insights into information distortion, highlighting potential trends that can be further explored in multicenter studies to strengthen generalizability. Furthermore, the findings were based on participants’ self-reported experiences. Because the interviews explored ethically sensitive documentation practices, participants may have underreported or minimized problematic behaviors due to social desirability bias, despite assurances of confidentiality. Their willingness to disclose such practices may also have been influenced by organizational culture and workplace norms. In addition, this study relied solely on interview data and did not incorporate methodological triangulation, such as direct observation or document analysis. Future research using multiple data sources could provide a more comprehensive understanding of information distortion in ENRs. Finally, all participants were female, and some were purposively recruited for their informatics-related experience. While these participants provided rich insights into electronic nursing documentation, their perspectives may differ from those of nurses without such experience, potentially limiting the applicability of the findings to the broader nursing population. Nevertheless, given the predominance of female nurses in the Chinese nursing workforce, the lack of male participants was difficult to avoid. Moreover, we included nurses with limited informatics-related experience, and purposive sampling was adopted to maximize variation in informatics experience and departmental backgrounds, thereby enriching the breadth and depth of the data.

### Conclusions

This research identified 4 main categories of information distortion manifestations in ENRs and 3 categories of their potential consequences. These findings underscore the importance of raising awareness of information distortion in health care data. Ethics training, monitoring mechanisms, standardized documentation guidelines, and technology-enabled solutions are recommended to address this issue. Further research is needed to analyze the contributing factors and develop effective countermeasures to improve the quality and reliability of nursing records.

## Supplementary material

10.2196/96679 Multimedia Appendix 1 Topic guide.

10.2196/96679 Checklist 1 COREQ checklist.

## Abbreviations

COREQ: Consolidated Criteria for Reporting Qualitative Research
EMR: electronic medical record
ENR: electronic nursing record
