# Supplementary material for: Manifestations and Potential Consequences of Information Distortion in Electronic Nursing Records: Qualitative Study
Source: JMIR Nurs. 2026 Aug 26;9:e96679. doi: 10.2196/96679 (PMC13509441; doi:10.2196/96679)
Supplement: Supplementary file 1 [file nursing-v9-e96679-s001.docx]

Topic Guide

***Introduction questions***

1. How long have you been working at this hospital?
2. Have you ever worked at another hospital before? Is so, in which care unit?

***Experiences***

1. Can you share any instances from your work where nursing records did not match reality?
a. When did that occur?
b. How did it happen?
2. In which nursing context do you believe information distortion problems typically occur?
a. How about in nursing assessments?
b. How about in nursing diagnoses?
c. How about in nursing care plans?
d. How about in nursing implementations?
e. How about in nursing evaluations?
f. Are there other nursing activities where you observe these issues?
3. Have you ever noticed your colleagues experiencing this phenomenon?
a. In what situations do your colleagues most often encounter information distortion issues?
b. Are there differences between new nurses and experienced nurses regarding these issues?

***Consequences***

1. What do you think are the implications of this phenomenon?
a. How do you believe it will affect you personally?
b. What implications do you think it will have on the nursing process?
c. What implications do you think it will have for patients?
d. What implications do you think it will have in other areas?
2. Do you think it is necessary to minimize this phenomenon as much as possible?

***Closing statements***

1. Is there anything else you would like to share that I haven’t asked you about yet?
